# Survival rate of cervical cancer: a five year review at a Major Teaching Hospital in Ghana, West Africa

**DOI:** 10.3332/ecancer.2024.1663

**Published:** 2024-02-01

**Authors:** Joseph Daniels, Kwesi Asante, Judith Naa Odey Tackie, Kofi Adesi Kyei

**Affiliations:** 1 National Radiotherapy, Oncology and Nuclear Medicine Centre, Korle Bu Teaching Hospital, Accra, Ghana; 2 Department of Radiography, University of Ghana, Accra, Ghana; a https://orcid.org/0000-0002-1466-150X

**Keywords:** cervical cancer, 5-year survival, cancer mortality, cancer survival, pap smear, cancer screening

## Abstract

Cervical cancer (CC) is one of the leading causes of cancer-related deaths among females in Ghana. Despite the magnitude of the public health challenge posed by CC in Ghana, survival data as well as reported incidence and mortality rates are primarily based on studies conducted in the capital city of the country. Even though age at diagnosis is known to affect the overall survival of CC patients, the role of this factor in the prognosis of CC patients in Ghana has not been sufficiently explored. The aim of this study was to determine the 5-year survival rate of Ghanaian woman treated for CC at a large tertiary healthcare facility in Ghana. This research was a single-institution-based quantitative retrospective cohort study conducted among patients with histopathologically confirmed CC. Clinical and socio-demographic data were retrieved from patients’ medical records. Data analysis was done using the Statistical Package for the Social Sciences software version 23. Kaplan Meier curves were used to present the survival rates and median survival time. The peak age at diagnosis was between 45 and 80 years with the modal age group of patients between 75 and 80 years. The mean age at diagnosis was 63.3 ± 15.7 years ranging from 27 to 104 years. The overall survival rates at 1, 3 and 5 years were 76.5%, 51.5% and 32.4%, respectively. The median survival time was 65.8 months. Age < 50 years was associated with higher survival estimates than age >50 years. The 5-year overall survival rate of CC patients reported in this study (32.4%) is relatively low compared with countries in the developed world but like previous reports at other healthcare facilities in Ghana as well as in other underdeveloped countries.

## Introduction

Cervical cancer (CC) is the fourth most frequently diagnosed cancer among adult females worldwide, and second among women between the ages of 15 and 44 years [1]. Notably, 80%–85% of all new cases occur in developing and resource-limited countries [2]. The drastic difference between the CC burden in developed countries and resource-limited settings is a reflection of the absence of widespread efficient screening for CC in the latter region. Even though the incidence of CC is about 30% higher in African Americans than in whites, the mortality is twice as high [3]. About nine out of every ten CC deaths occur in under developed areas, and the average risk of death from CC before the age of 75 is three times higher in the less developed regions of the world than in more developed countries [4]. Collectively, low- and middle-income countries are expected to account for more than 95% of CC deaths by 2030 [5].

The incidence and mortality rates of CC have fallen drastically in developed nations such as the United States of America, which observed a 70% decrease from 1955 to 1992 [6]. However, CC survival rates have remained unchanged or even risen in many LMICs [7]. In East Africa and South Asia, CC remains one of the most common cancers in women, and the number one cause of death [8]. Similarly, West Africa has recorded more than three times the global CC mortality rates (18.5 age standardised rate per 10,000). In Ghana, CC is the second most common cancer among women, with an estimated 3,052 new cases and 1,556 deaths in 2012. The World Health Organisation predicts that by the year 2025, 5,000 new cases of CC and 3,361 CC deaths will occur annually in Ghana [2]. The commonest etiological factor for CC is persistent infection with high-risk human papillomavirus strains, specifically type 16 and 18 [9].

Despite the magnitude of the public health challenge posed by CC in Ghana, survival data as well as reported incidence and mortality rates are primarily based on studies conducted in the capital city of the country. There are three large hospital-based cancer registries located in three publicly funded tertiary healthcare facilities in Ghana, namely Komfo Anokye Teaching Hospital (KATH), Cape Coast Teaching Hospital and the Korle Bu Teaching Hospital (KBTH). The Oncology Directorate and Radiotherapy centre at KATH and KBTH respectively, serve as the main cancer referral centres in Ghana where patients with malignant disease are diagnosed and treated with a combination of chemotherapy and radiotherapy as well as other modalities as necessary. KATH receives referrals of patients with CC from all over the country as well as neighbouring West African countries such as Togo, Sierra Leone and Burkina Faso. Even though age at diagnosis is known to affect the overall survival of CC patients [10], the role of this factor in the prognosis of CC patients in Ghana has not been sufficiently explored [11]. The aim of this study was to determine the 5-year survival rate of women treated for CC at a major teaching hospital in Ghana and to explore the association of patients’ ethnicity and age at the time of diagnosis with overall survival.

## Methods

This research was a single-institution-based quantitative retrospective cohort study conducted among patients with histopathologically confirmed CC who received treatment in 2012. The study was conducted at the KATH, a 1,200-bed tertiary healthcare facility in the Ashanti region of Ghana. It is the second largest hospital in the country and receives referrals from 13 out of the 16 administrative regions of Ghana. The study included only patients who completed the recommended treatment for CC at the study site. Clinical and socio-demographic data were retrieved from patients’ medical records including patients’ contact telephone numbers, the date of diagnosis, age at diagnosis, and outcomes of treatment. The specific dates of commencement and completion of full treatment was also documented. Patients or their next of kin were contacted via telephony to elicit information about their clinical status and mortality i.e., whether dead or alive. A total of 105 patients with a histopathologically confirmed diagnosis of CC were diagnosed at the study site in 2012. However, only 88 patients completed full definitive treatment with chemoradiation followed by brachytherapy. Even though contact information was available for all the patients, 15 contact numbers turned out to be out of service for which reason these specific patients could not be reached on phone for possible recruitment into the study. Out of the remaining patients, 5 declined to provide consent for participation in the study as a result of which a total of 68 patients were included in the study. The exact dates of death of patients were tabulated in a data collection spreadsheet. Data analysis was done using the Statistical Package for the Social Sciences software version 23. Survival analysis was done with stratification by age and menopausal status. Kaplan Meier analysis was conducted to evaluate the overall survival rates and median survival time of the patients. Patients’ data was completely anonymized and their confidentiality maintained. Participants were informed about their right to refrain from participating in the study at the very beginning of the telephone interview. Only eligible patients who provided informed consent were recruited in the study. For patients who were deceased, informed consent was obtained from their next of kin. Ethical approval was obtained from the Ethical and Protocol Review Committee of the School of Biomedical and Allied Health Sciences, College of Health Sciences, University of Ghana, Legon prior to commencement of the study.

## Results

**Figure 1.**
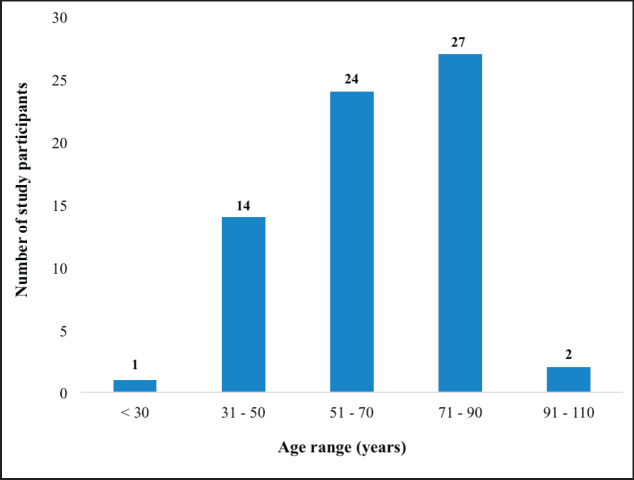
Distribution of CC patients based on their age at the time of diagnosis.

In total, 68 patients were recruited into the study. Even though data were retrieved for 88 CC patients who received full treatment during the study period, 20 (22.7%) either declined consent or had been lost to follow up and were unreachable for the provision of consent to participate in this study. The median age at diagnosis was 65 years ranging from 27 to 104 years. Figure 1 illustrates the age distribution of the participants in the study. The mean age of the patients at the time of diagnosis was 63.3 ± 15.7 years.

**Figure 2.**
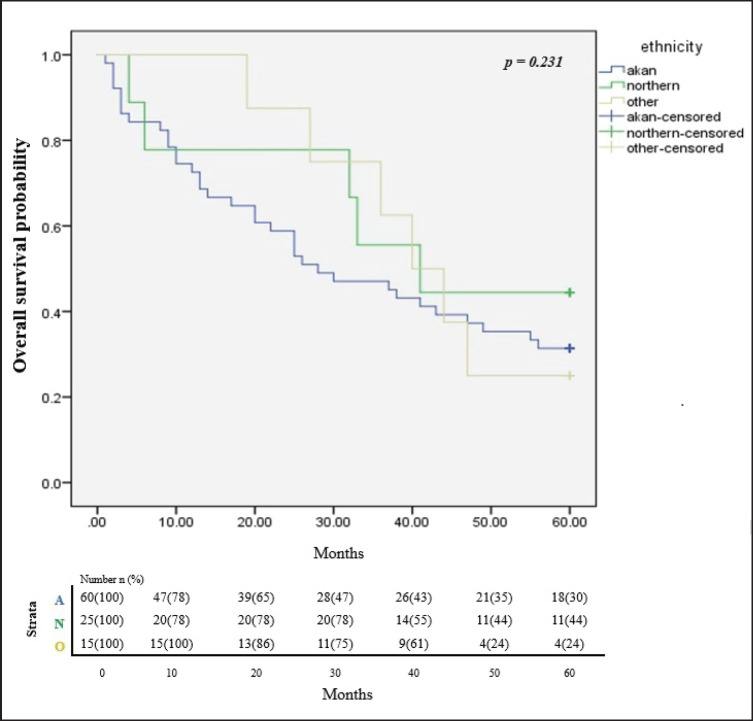
Cumulative overall survival estimates based on ethnicity. A = Akan ethnicity, N = Northern ethnicity, O = Other ethnicities.

**Figure 3.**
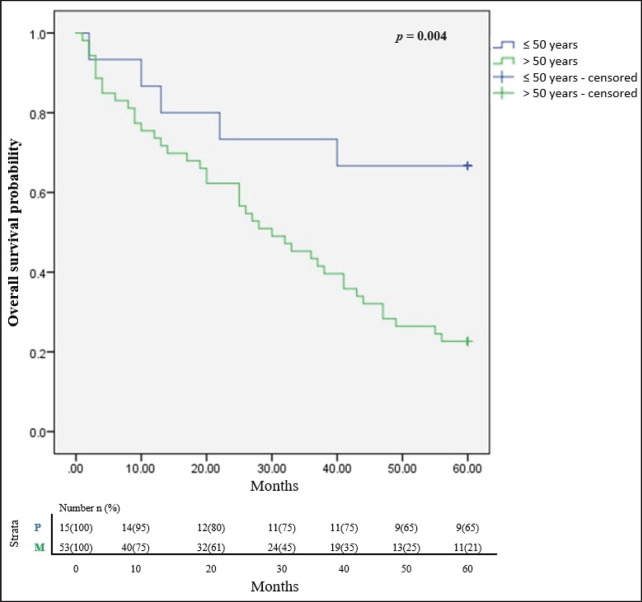
Cumulative overall survival estimates based on age ≤50 and >50 years. *p* = Age ≤50 years, M = Age >50 years.

In all, only 32.4% of the participants were still alive after 5 years of follow up since diagnosis and successful completion of their full treatment. The overall survival rates at 1, 3 and 5 years were 76.5%, 51.5% and 32.4% respectively. The median survival time was 65.8 months. Figure 2 illustrates the Kaplan Meier estimates showing the cumulative survival probabilities of patients based on their ethnicity whereas Figure 3 illustrates overall survival rates based on the age groups of the patients whether younger or older than 50 years. Northern ethnicity was associated with the longest overall survival estimates followed by Akan ethnicity. Patient’s age ≤50 years was associated with higher survival estimates than age >50 years (*p* = 0.004).

## Discussion

This study demonstrates a 5-year CC overall survival rate of 32.4% which is slightly lower than the previously reported survival rate of 41.3% in the capital city of Ghana. That study was conducted at the National Center for Radiotherapy, Oncology and Nuclear Medicine at KBTH in Accra and showed that out of a total of 100 CC patients diagnosed in 2012, 43.1% were still alive 5 years after the completion of their treatment [11]. Whereas KATH is the second largest tertiary hospital in Ghana, KBTH is the largest hospital in the entire West African subregion and the fourth largest on the African continent. Survival rates of CC for each country differ throughout the world and are intricately linked with each country’s development status. Notably, the 5-year survival rates of CC patients are generally high in developed countries but low in LMICs. Asian countries such as China and Thailand have reported 5-year survival rates exceeding 50% [12]. Similarly, the reported 5-year CC survival rate in England and Wales is 67.4% [13]. Other developed countries such as France, Japan and Australia have reported much higher 5-year survival rates of 70%, 71.5% and 73.6%, respectively [14]. In contrast, LMICs in Africa such as Gambia and Uganda have reported 5-year CC survival rates ≤25% [15].

A previous study in Ghana reported that among the myriad of reasons for Ghanaians having a low 5-year CC survival rate is their peculiar health-seeking behaviour. Many tend to have a strong inclination for initial traditional treatment and self-medication in lieu of immediate modern treatment irrespective of the availability of the latter [16]. It has also been reported that Ghanaians tend to seek treatment at a later stage of cancer and usually present at treatment centres with large tumours that are less amenable to curative therapy [11]. A similar challenge has been observed among Ghanaians patients with breast cancer [17]. Lower educational attainment, older age, obesity, smoking, and poverty are independently related to lower rates of CC screening [18]. Additional probable underlying factors associated with the poor survival of CC patients in Ghana include the limited attention given to the disease, poor awareness, late diagnosis, missed diagnosis and misdiagnosis as well as the high cost of comprehensive cancer treatment. Several studies have shown that the incidence of CC, as well as its survival and mortality, vary with ethnicity and socio-economic status [19].

Although CC causes more mortality than breast cancer among Ghanaian women, the latter is more frequently diagnosed among women than the former. CC receives limited attention with most cancer-related educational and public health campaigns directed towards other cancers, especially female breast cancer. In Ghana and around the world, January has been set aside as the CC awareness month. However, insufficient effort, time and resources are dedicated to the dissemination of information and organisation of screening programs during the CC awareness month. The relatively low level of awareness of CC indirectly leads to late presentation with locally advanced or metastatic disease which portends for poor overall survival [16]. By providing comprehensive and accessible education on CC, communities can empower individuals to make informed decisions about their health, encourage early detection, and ultimately reduce the prevalence and impact of this disease.

Again, in Ghana, treatment for CC is not covered by the National Health Insurance Scheme (NHIS) which is the most popular insurance scheme among the populace. As a result, CC patients, many of whom are socioeconomically underprivileged have to cover the cost of their diagnosis, clinical workup and treatment entirely out of pocket. Those who are unable to afford treatment usually end up resorting to ineffective unorthodox remedies or remain without appropriate care. Expanding the scope of medical conditions covered by the NHIS to include CC will therefore go a long way to reduce the financial burden on the affected patients and their relatives.

Another factor influencing the survival rate of CC patients in Ghana is limited access to timely and efficient health care even after the appropriate diagnosis has been made. Most patients eventually present to oncologists with advanced disease which requires the use of chemotherapy combined with external beam radiotherapy and brachytherapy for curative treatment when possible or otherwise for palliative management. Ghana currently has only three radiation therapy centres, two of which are located in the capital city (Accra) in the southern part of the country whereas the third is located in the middle part of the country where this study was conducted. Patients often have to travel over long distances from other regions of the country to access these treatment facilities. The cost and inconvenience envisaged might be prohibitive or even unacceptable to some patients resulting in failure and/or refusal to receive adequate and/or appropriate oncological care.

Over the years, the burden of high CC incidence and mortality has shifted to less developed countries [19]. The majority of CC now occurs in developing countries and medically underserved populations due to the lack of Papanicolaou smear screening [20]. The continuing global demographic and epidemiologic transition signal an ever-increasing cancer burden over the next decades, particularly in LMICs, with over 20 million new cancer cases expected annually as early as 2025 [21].

Pap smear screening has led to a significant reduction in the incidence of CC in the United States of America. Even though this test has a sensitivity of 55%–80% [22] serial screening as prescribed by international clinical guidelines is important. Regular screening can dramatically reduce the incidence of CC. Conversely, poor survival and low quality of life occur when the cancer is detected at later stages of the disease. The chances of survival for patients diagnosed with stage IA is nearly 100% compared to patients diagnosed with stage IVB whose chances of survival are about 20% [23]. Screening for cytologic abnormality by Pap smear is documented to have led to significant reduction in the incidence of CC in the United States [24, 25]. Similar results can be achieved in Ghana if active attempts are made to expand the availability, accessibility and affordability of screening cervices for CC in the country.

## Limitations

Even though there are only three cancer radiotherapy treatment centres in Ghana where patients can be fully treated with radiotherapy and chemotherapy, not all CC patients are referred to these facilities. Patients with incurable disease are the least likely to be referred for expensive treatment with radiation and/or chemotherapy. This study was conducted at a single tertiary healthcare facility in Ghana and may not necessarily reflect survival rates of CC patients treated throughout the entire country.

## Recommendations

Even though the rollout of human papillomavirus vaccines holds the promise of significantly reducing the incidence of CC in Ghana, issues of cost, affordability and accessibility are yet to be addressed in Ghana. Health institutions and key stakeholders must therefore continue and increase the effort to make services related to the screening, diagnosis and treatment of CC in Ghana affordable for women at risk of the disease in Ghana.

## Conclusion

The 5-year overall survival rate of CC patients managed at KATH is 32.4% which is relatively low compared with countries in the developed world but similar to previous reports at other healthcare facilities in Ghana as well as in other underdeveloped countries.

## Conflicts of interest

The authors declare no competing interest.

## Funding

This study did not receive any specific funding support from funding agencies in the public, commercial, or not-for-profit sectors.

## Data availability

The data used to support the findings of this study are available from the corresponding author upon reasonable request.
